# mTORC1 Enhances Early Phase Ribosome Processivity

**DOI:** 10.3389/fmolb.2020.00117

**Published:** 2020-06-19

**Authors:** Erin An, Kyle Friend

**Affiliations:** Department of Chemistry and Biochemistry, Washington and Lee University, Lexington, VA, United States

**Keywords:** ribosome processivity, translation elongation, mTOR, ribosome density index, ribosome profiling

## Abstract

During translation elongation, the ribosome serially adds amino acids to a growing polypeptide over many rounds of catalysis. The ribosome remains bound to mRNAs over these multiple catalytic cycles, requiring high processivity. Despite its importance to translation, relatively little is known about how mRNA sequences or signaling pathways might enhance or reduce ribosome processivity. Here, we describe a metric for ribosome processivity, the ribosome density index (RDI), which is readily calculated from ribosomal profiling data. We show that ribosome processivity is not strongly influenced by open-reading frame (ORF) length or codon optimality. However, we do observe that ribosome processivity exists in two phases and that the early phase of ribosome processivity is enhanced by mTORC1, a key translational regulator. By showing that ribosome processivity is regulated, our findings suggest an additional layer of control that the cell can exert to govern gene expression.

## Introduction

Translational output from an mRNA helps govern protein expression. Over the past decade, ribosomal profiling has been used to intensively study translational output since it measures ribosomal density on all mRNAs (Ingolia et al., 2009, 2012). When quantified, mRNA levels and ribosomal densities serve as excellent predictors of protein levels, better than measurements of mRNA levels alone (Ingolia et al., 2009). Beyond quantitating the numbers of ribosomes bound to an mRNA, ribosomal profiling can also give insight into translational dynamics during elongation.

Eukaryotic translation is a multi-step process including initiation and elongation phases, and translation initiation is tightly controlled in the cell (reviewed in Sonenberg and Hinnebusch, 2009; Roux and Topisirovic, 2018). Far less is known about translational control during elongation, but some evidence exists. During cellular heat-shock, ribosomes pause on mRNAs within the ORF as nascent proteins first emerge from the ribosome, likely allowing misfolded proteins to refold before translation can resume (Shalgi et al., 2013). This mechanism is reminiscent of stalled ribosomes that accumulate on mRNAs encoding secreted or membrane proteins. With these mRNAs, nascent polypeptide is bound to the signal recognition particle (SRP) which freezes translation until mRNA can dock with the endoplasmic reticulum (Walter and Blobel, 1981). In addition, certain RNA-binding proteins such as FMRP (fragile X mental retardation protein) and Pumilio likely inhibit translation elongation (Darnell et al., 2011; Friend et al., 2012). Even well-documented initiation regulators such as mTORC1 can influence translation elongation (Faller et al., 2015). Faller et al. showed that mTORC1 regulates translation elongation via the mTORC1–S6K–eEF2 kinase axis. So, although initiation is well-documented as a regulatory checkpoint, translation elongation is also regulated.

The ribosome is a highly processive enzyme, staying bound to mRNAs over many catalytic cycles. In the examples outlined above, ribosome stalling has been observed, but could ribosome processivity also be regulated? Despite a need for processivity, not all ribosomes are fully processive. In *E. coli*, ribosomes fail to synthesize full-length polypeptides when ORF length increases (Tsung et al., 1989). In yeast, there is unexpectedly low ribosome density on longer ORFs (Arava et al., 2003). Also, ORF 5′ ends have more ribosome density compared to 3′ ends which may reflect a difference in processivity but also may be due to slower translation elongation rates at the ORF 5′ end (Ingolia et al., 2009; Shah et al., 2013). In mammals, estimates are that ~30% of newly produced peptides are degraded (Wheatley and Inglis, 1985; Schubert et al., 2000), and co-translational peptide degradation has been observed (Turner and Varshavsky, 2000; Chuang et al., 2005). More recently, tandem luciferase reporter mRNAs were used to show that the ribosomes translating a reporter mRNA become more processive over time (Bonderoff and Lloyd, 2010). For viral mRNAs, ribosomes often undergo programmed frameshifting during translation at structured mRNA sequences (Caliskan et al., 2015). Interestingly, at these difficult-to-translate sequences, spontaneously aborted protein products are readily observed arguing that ribosomes are dissociating from the mRNA without encountering a stop codon (Lopinski et al., 2000; Kontos et al., 2001).

Currently missing within the field of translational research is a quantitative method for characterizing ribosome processivity. Here, we report the creation and use of the RDI, which is readily calculated from ribosomal profiling data. Using RDI values calculated from mTORC1-inhibited cells, we show that many mRNAs previously demonstrated to be regulated by mTORC1 during initiation are also regulated during early translation elongation, especially ribosomal protein-encoding mRNAs. mTORC1 activity stabilizes mRNA-bound ribosomes early in translation, preventing ribosome drop-off. We describe regulated ribosome processivity as an additional layer of translational control.

## Materials and Methods

### Ribosome Density Calculations and RDI

Raw FASTQ files were downloaded from the Gene Expression Omnibus (GSE36892; Thoreen et al., 2012). We first aligned reads to mouse rRNA sequences using the Bowtie algorithm (Langmead et al., 2009), removing rRNA reads. For ribosome-protected fragment (RPF) alignment, we set seed length to 15 nucleotides and aligned to the mouse transcriptome (from the mm 10 genomic assembly). Only aligned reads were further processed. For total mRNA alignments, we similarly mapped these to the mouse transcriptome.

To construct information on ribosome density, a custom script summed read depth over three nucleotide codons. We then removed lowly-translated mRNAs (i.e., those with >25% of codons that had zero ribosome density in any one biological replicate). For the remaining mRNAs, we calculated ribosome densities by dividing RPF read depth by total mRNA TPM values. We then used this trimmed subset of mRNAs for subsequent analysis (~2,300 mRNAs). At this point, two Control sample profiles, and two Torin1-treated sample profiles remained.

To calculate RDI, we used a custom script to multiply the ribosome density at each codon by the codon number, calculating the sum. That sum was then divided by the total ribosome density across the transcript multiplied by the total length of the transcript. We verified that RDI values centered close to 0.5 (0.48 and 0.46, respectively).

The codon adaptation index was calculated as described (Sharp and Li, 1987) using the RIKEN mouse genome codon abundances.

### Simulations

**Figure 1:**
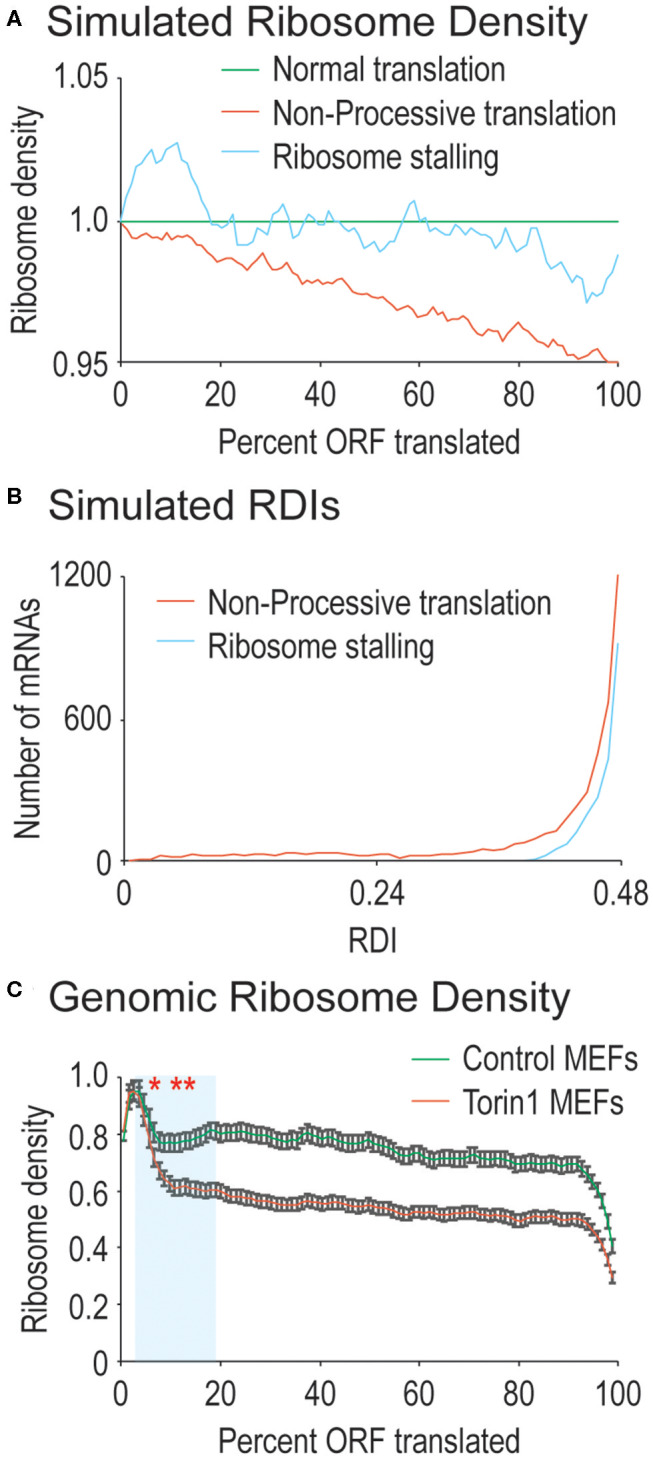
Ribosome processivity has two phases. **(A)** We simulated how ribosome density would be affected by incomplete ribosome processivity or ribosome stalling. Poor ribosome processivity was modeled by assigning a probability that a translating ribosome would dissociate from the mRNA (here with a probability of 1 in 40 elongation events). Stalling was modeled by increasing ribosome density on the 5′ side of a stall site and decreasing density on the 3′ side of a stall site (here with an exaggerated 10-fold higher density before the stall compared to after the stall). **(B)** We calculated RDI values for every mRNA after running our simulations. Non-processive translation results in lower RDI values compared to ribosome stalling. Note that RDIs between 0.48 and 0.50 are not shown, but that more mRNAs have these RDI values in the ribosome stalling simulation. **(C)** Shown are actual ribosome densities for all translated mRNAs in the genome normalized to ORF length (i.e., codon 200 in a 1,000 codon-long ORF is considered 20%). mRNAs were derived from cells grown in the absence (Control) or presence of Torin1. In both cases, the curves exist in two phases, the first phase highlighted in blue has a consistently lower slope after Torin1 treatment. The results are significantly different (Mann-Whitney *U*-test, ^*^and to the right, *p* < 0.01, ^**^and to the right, *p* < 1 e^−10^). Note that 99% confidence intervals are shown with error bars.

To simulate differences in ribosome processivity, we first calculated average ribosome density for every mRNA in the genome using the data above. Then, we created a simulated pool of mRNAs that had uniform and even ribosome density along their open-reading frames. We modeled progressively-increasing rates of ribosome drop-off by setting probabilities that ribosomes would spontaneously abort translation as they transited mRNAs. The data shown in Figure 1A are from simulations where 1 in 40 translation events resulted in aborted translation.

To simulate ribosome stalling, we again used the simulated pool of mRNAs from above. To each mRNA, we introduced one random stall site by modeling the increased ribosome density that would be found on the 5′ side of the stall site as well as the decreased ribosome density that would be found on the 3′ side of the stall site. The data presented in Figure 1A represent stall sites with 10-fold higher density on the 5′ side of the site compared to the 3′ side of the stall site.

### Statistical Analyses

Empirical *p*-values were calculated using a Monte Carlo method with random subsampling of all RDIs, comparing Control-treated and Torin1-treated samples. Known mTORC1-regulated mRNAs (Thoreen et al., 2012) were then compared against an equal number of randomly-subsampled mRNAs using the difference in RDI between Control-treated and Torin1-treated mRNAs. *P*-value was calculated by repeating this subsampling 10,000 times.

For analyses involving ribosome densities along the mRNA (either as a fraction of the ORF or by codon), we treated each region of the mRNA independently and used a Mann-Whitney *U*-test to calculate reported *p*-values.

All custom scripts were written in Python and are available on GitHub (https://github.com/Kyle-Friend/Processivity).

## Results

### The Ribosome Density Index (RDI)

Ribosomal profiling can be used to calculate ribosomal density across mRNA ORFs (Ingolia et al., 2009, 2012). Since the ribosome binds mRNAs during translation and protects them from nuclease treatment, these ribosome-protected fragments, or RPFs, can be used to infer the number of ribosomes at specific positions along an mRNA. We sought to convert these data into a metric for ribosome processivity.

For completely processive ribosomes, ribosomal density should be uniform across the ORF since all ribosomes that initiate translation will reach the stop codon, leaving average density midway through the ORF. For ribosomes that lack processivity, translation initiation should create higher ribosomal density at the 5′ end of the ORF that tapers toward the ORF 3′ end as ribosomes spontaneously abort translation, skewing average density toward the ORF 5′ end (see simulated data in Figure 1A). In addition to processivity, ribosomes are also known to stall (reviewed in Wilson et al., 2016). We also simulated ribosome stalling to see how it would affect ribosomal density across mRNA ORFs. At the position of a stall, ribosome density is expected to accumulate on the 5′ side and to decrease on the 3′ side. However, there should be a roughly even density of ribosomes across mRNA ORFs except at the stall site (see simulated data in Figure 1A). When examined at a genomic level, since stall sites are distributed across mRNAs, there is no obvious difference between simulated mRNAs with stalled ribosomes vs. those that do not contain stalling. Therefore, we created a metric to quantify differences in ribosome density along mRNA ORFs with the following equation:

$$\begin{array}{l} RDI=\frac{\sum_{i=1}^{n}\left(i*RPF_{i}\right)}{n\sum_{i=1}^{n}RPF_{i}}\; \end{array}$$

Here, RPF_i_ is the normalized number of RPFs at codon number i, and n is the total number of codons within the mRNA ORF. This calculation creates the RDI, where 0 < RDI <1. RDI values <0.5 have average ribosome density skewed toward the 5′ end of the ORF. It is expected that the majority of mRNAs will have an RDI of ~0.5 since ribosomes should be roughly evenly distributed across ORFs.

We next explored how RDI would change using the simulated data outlined above. Ribosome density can accumulate due to slowed translation elongation rates at pause sites (Ingolia et al., 2009). After a pause site, ribosome density would decrease making it difficult to distinguish between ribosome drop-off and ribosome stalling. We calculated RDIs for poorly processive ribosomes as well as strongly stalled ribosomes to see how RDI would be affected. Stalling results in slight lowering of RDI, whereas a loss in ribosome processivity has a stronger effect (Figure 1B, average RDI from stalling is 0.49, from low processivity, 0.45). It is important to note that other effects on translation elongation would not be detected with RDI, such as differences in ribosome elongation rates across the ORF. These would be expected to either increase or decrease overall ribosome density, but not necessarily the distribution of ribosomes on mRNAs. These simulated data argue for the use of RDI as a metric for ribosome processivity.

### mTORC1 Regulates Ribosome Processivity

We next investigated ribosome processivity in a mammalian system where translation elongation should be regulated. Previously, ribosomal profiling experiments were performed on mouse embryonic fibroblasts (MEFs) that were grown in the absence or presence of the highly-specific mTORC1 inhibitor, Torin1 (Thoreen et al., 2012). At the time of this work, it was not established that mTORC1 would regulate translation elongation, but rather, mTORC1 inhibition of translation initiation was well-established. Later, the mTORC1—S6K—eEF2 kinase axis was established as a mechanism whereby mTORC1 could influence translation elongation (Faller et al., 2015). mTORC1 has been intensively studied, and mTORC1 inhibition removes target mRNAs from polyribosome and monoribosome fractions which is inconsistent with ribosomal stalling mechanisms (Jefferies et al., 1994; Thoreen et al., 2012; Faller et al., 2015). Therefore, these earlier experiments were ideal for querying specific effects on ribosome processivity and the use of RDI.

We first calculated RDI values for mRNAs derived from MEFs grown in the presence or absence of Torin1. We aligned RPFs against the mouse transcriptome, and we surveyed translated mRNAs for decreasing ribosome density across the ORF which would indicate defects in ribosome processivity (Figure 1C). For RPFs isolated from control MEFs, we did observe a relatively steady decrease in ribosome density across ORFs, leading to an average RDI of 0.48. In previous ribosome profiling experiments, higher ribosome density has been observed at the 5′ end of the ORF attributed to slowed translation elongation rates, and our data are consistent with that observation (Ingolia et al., 2009). When cells were treated with Torin1, the trends were strikingly different (Figure 1C). Now the relatively steady decrease in ribosome density split into two phases, an early phase where Torin1-treated samples diverged from the control and the second with a more gradual decrease in ribosome density similar to ribosome densities from control cells. Given the ribosome densities observed, mRNAs from Torin1-treated cells had a downward shift in average RDI values to 0.46. These data argue that ribosome processivity may exist in two phases, the first of which is potentially dependent on mTORC1 activity.

### Codon Optimality and ORF Length Minimally Influence Ribosome Processivity

**Figure 2:**
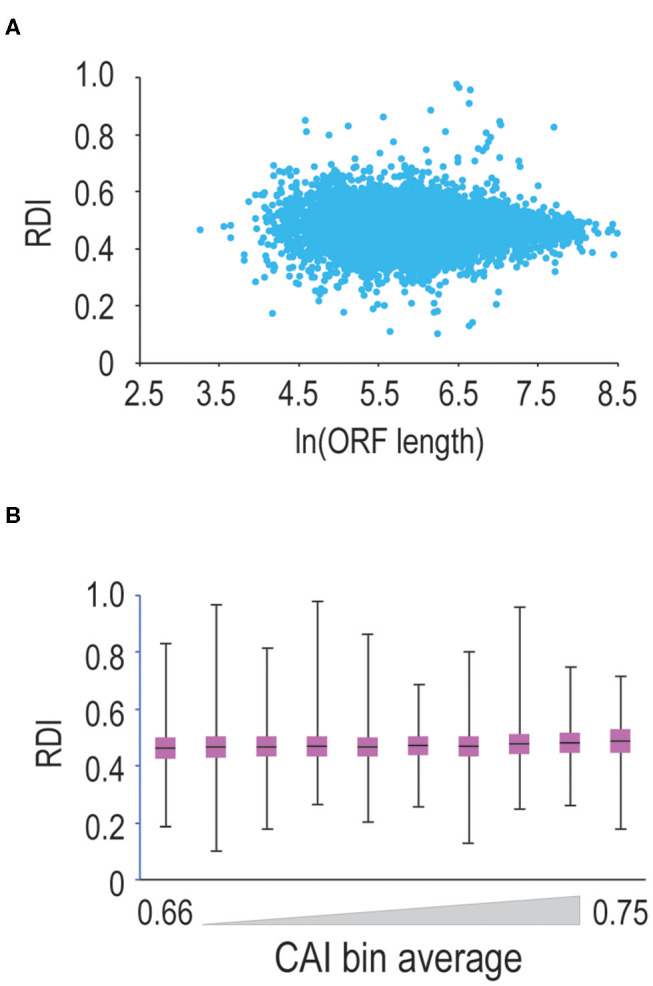
ORF length and codon optimality do not significantly affect ribosome processivity. **(A)** We calculated RDI values for every mRNA in the genome and then plotted RDI values vs. the natural log of ORF length. As ORF length increases, we do not observe a significant difference in RDI values. **(B)** We calculated CAI values for every translated mRNA in the mouse genome and sorted mRNAs from lowest to highest CAI values. We binned mRNAs into ten groups and then compared RDI values. Shown is a box-and-whisker plot of RDI values in these bins. There is a modest positive correlation between RDI values and CAI values, but the correlation is not significant.

Translation elongation can be influenced by a number of factors, including codon bias (Varenne et al., 1984), and potentially, open-reading frame length (Arava et al., 2003). Therefore, we continued our analysis by querying ORF-length vs. RDI. Shown in Figure 2A are data comparing ORF-length to RDI. Mouse ORFs within our dataset are 520 codons long on average, leading to clustering toward the middle of the scatterplot. Moving toward longer or shorter ORFs, we do not observe significant changes in RDI, arguing that ORF length does not strongly correlate with ribosome processivity.

Next, we asked whether codon optimality influences ribosome processivity. Since the genetic code is degenerate, many amino acids are encoded by multiple codons. Some synonymous codons are more prevalent than others, and the most commonly-used codons are decoded by more abundant tRNAs making these codons more optimal for translation (Varenne et al., 1984). So, we tested whether codon optimality influences ribosome processivity. We first calculated CAI (codon adaptation index; Sharp and Li, 1987) values for every mRNA in our dataset. Note that mRNAs with less optimal codons are characterized by lower CAI values. We then sorted and binned mRNAs by CAI values and calculated RDI values for these binned mRNAs (Figure 2B). We observe that as CAI values increase, there is a very modest trend toward higher RDI values (note that 0.5 corresponds to complete ribosome processivity). These changes are not significant, arguing that codon optimality minimally affects ribosome processivity, although we cannot rule out the possibility that short stretches of sub-optimal codons may promote ribosome drop-off.

### mTORC1 Regulates Early Phase Ribosome Processivity

**Figure 3:**
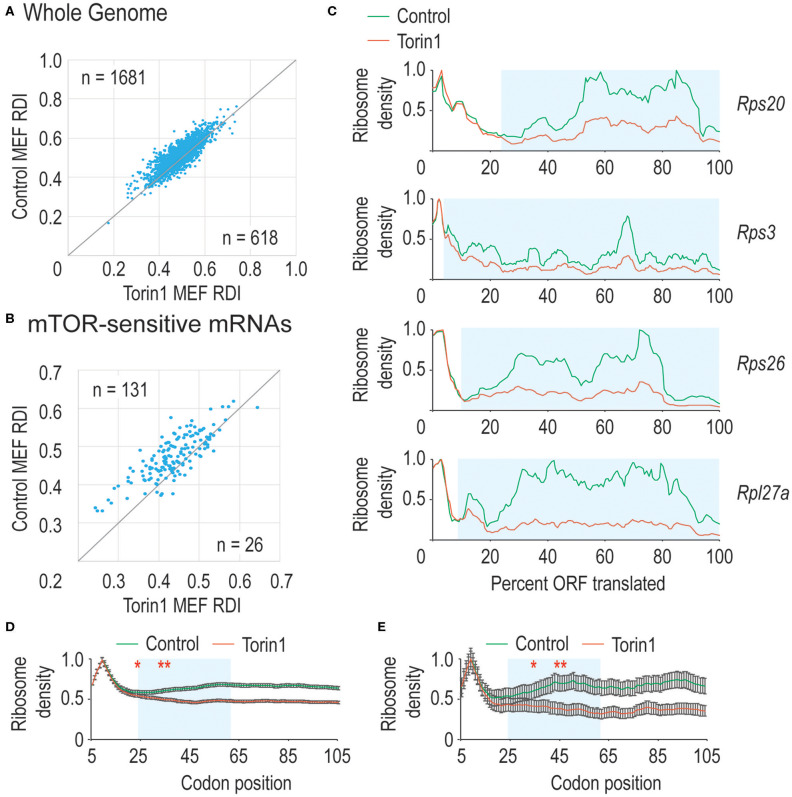
mTORC1 is necessary for early phase ribosome processivity. **(A)** We calculated RDI values for all translated mRNAs from cells grown in the absence (Control MEFs) or presence (Torin1 MEFs) of Torin1. RDIs for the two conditions were plotted, with the number of mRNAs in each half of the plot indicated. The higher value in the upper left corner indicates that the majority of mRNAs have higher RDI in control cells, indicating that mTORC1 increases ribosome processivity for most mRNAs (Mann-Whitney *U*-test, *p* = 9.2 e^−32^). **(B)** We repeated the analysis in part A, but focused instead on known mTORC1-regulated mRNAs. These include many mRNAs encoding ribosomal proteins and other translation factors. As above, mTORC1 is important for ribosome processivity on these mRNAs (empirical *p* < 0.001). **(C)** We plot ribosome densities for a limited set of mTORC1-regulated mRNAs. In all cases, we normalized the data to the codon with highest density. In every case, the ribosome densities overlap regardless of whether mTORC1 was active in the 5′ region of the ORF, but then the curves diverge, with mRNAs from Torin1-treated cells having lower density (Mann-Whitney *U*-test, blue boxed regions give *p*-values of 3.9 e^−13^, 2.9 e^−46^, 7.5 e^−12^, and 1.1 e^−32^). **(D,E)** We repeated the analysis in (1C), but focused on translation one codon at a time. We analyzed a window from the 5th codon to the 105th codon (^*^note that sequence read mapping was variable at the start codon). As above, mRNAs from Torin1-treated cells initially have high ribosomal density, but this rapidly decreases compared to mRNAs derived from control cells in **(D)**. Highlighted in blue is the region where the curves continue to diverge. This corresponds to codons 15–55. After that point, the spacing between the curves is equivalent. Similar results were observed with mTOR-sensitive mRNAs in **(E)**. The results are significant (Mann-Whitney *U*-test, ^*^and to the right, *p* < 0.01, ^**^and to the right, *p* < 1E-10). Note that 99% confidence intervals are shown with error bars.

In Figure 1C, we observed that mTORC1 boosted ribosome processivity, so we sought to explore this further. We compared RDI values for all mRNAs derived from MEFs grown in control conditions where mTORC1 activity should be high compared to Torin1-treated conditions where mTORC1 activity is reduced to see whether the trends we observed in Figure 1C were driven by translation on a limited set of mRNAs or translation on most mRNAs. By directly comparing RDI values for mRNAs from both conditions, we could ask how many mRNAs had lower RDI values in Torin1-treated cells compared to those in control cells. We observed that the majority of mRNAs had lower RDI values if they were derived from Torin1-treated MEFs (Figure 3A). Additionally, some mRNAs have high and low RDI values compared to the majority of mRNAs that have RDI values ~0.5; we include the identity of these mRNAs in Supplemental Table 1. We were unable to identify distinguishing features among these mRNAs, but an open question is why ribosome densities are so skewed among these mRNAs. These data are consistent with the expectation that mTORC1 influences bulk translation (Thoreen et al., 2012).

mTORC1 regulates translation of mRNAs encoding ribosomal proteins and translation factors as members of the TOP mRNAs (reviewed in Meyuhas, 2000). TOP mRNAs are characterized by a C immediately after the mRNA cap and highly structured, pyrimidine-rich 5′ UTRs. We next separated mTORC1 target mRNAs from the genomic dataset to analyze their RDI values. Under control conditions, these mRNAs had an average RDI of 0.49 which is marginally higher than that in the remaining genomic mRNAs. But when cells were cultured with Torin1, these mRNAs had a significantly lower average RDI of 0.45 (Figure 3B) which exceeded the more modest 2% decrease observed across the whole genome (empirical *p* < 0.001).

Previously, we observed that mTORC1 activity was required for early-phase ribosome processivity, so we analyzed ribosome densities on a limited set of mRNAs that had the largest decrease in ribosome processivity and were known mTORC1 targets. Shown in Figure 3C are data for four ribosomal protein-encoding mRNAs. For each mRNA, the normalized data largely overlap at the 5′ end of the ORF, diverging as translation proceeds. These data suggested that mTORC1 activity might be required over a narrow window early in translation elongation, so we zoomed in at the codon level to determine where mTORC1 might be required. Plotted in Figures 3D,E are data for all mRNAs as well as known mTORC1-sensitive mRNAs. Interestingly, there is a window from ~codon 15 until codon 55 over which ribosome processivity decreases on Torin1-treated cellular mRNAs. Given the importance of mTORC1 activity for translation initiation, it was interesting that no effect was observed before amino acid 15 (although it should be noted that read mapping to the start codon was variable making it difficult to analyze ribosome density directly at the start codon). Torin1 treatment has no effect later in translation elongation that we could observe. These experiments confirm that mTORC1 activity is required for early ribosome processivity and indicate that mTORC1 activity is required over a narrow window from codons ~15–55.

## Discussion

Here, we present the RDI, a tool to assess translation elongation dynamics. In simulations, we show that RDI is sensitive to differences in ribosome processivity, but less sensitive to ribosome stalling. In a mammalian system, we show that RDI is not strongly correlated with ORF length or codon optimality, but RDI responds to mTORC1 activity. mTORC1 is critical for early ribosome processivity in a narrow window, between codons ~15 and 55, but it is less critical later as ribosome density gradually decreases across the remainder of the ORF, consistent with ribosome drop-off and incomplete ribosome processivity. These findings provide insight into an unknown role for mTORC1 in ribosome processivity.

How might mTORC1 connect to ribosome processivity? Previous research has shown that nascent protein folding helps facilitate ribosome movement along the mRNA (Goldman et al., 2015; Nilsson et al., 2015). Where observed, that mechanism occurs when the ribosome is ~55 codons into the ORF. That mTORC1 activity seems to be required until ~55 codons have been translated would be consistent with this model, that the emerging peptide begins to fold and then helps push the ribosome forward. Prior to that event, mRNA ratcheting in the ribosome could be delayed, with an increased risk of ribosome drop-off.

Previous research has indicated that ribosome processivity is imperfect and that ribosome processivity can increase over time (Bonderoff and Lloyd, 2010). Our findings are consistent with that prior work, since we observe high levels of ribosome drop-off very early in translation. Ribosomes can form higher-order structures (Kopeina et al., 2008), and one model is that ribosome polymerization may contribute to higher processivity. Such a model requires many ribosomes to decorate an mRNA before they can become fully processive. It seems likely that the later phase of ribosome processivity connects to such a model where more highly processive ribosomes bind more distal parts of the ORF. In summary, we identify ribosome processivity as a regulated step of translation elongation.

## Data Availability Statement

The datasets used in this study can be found in the Gene Expression Omnibus (GSE36892; Thoreen et al., 2012).

## Author Contributions

EA and KF performed analyses. KF wrote the manuscript. All authors contributed to the article and approved the submitted version.

## Conflict of Interest

The authors declare that the research was conducted in the absence of any commercial or financial relationships that could be construed as a potential conflict of interest.
